# Structure-based antigenic epitope and PEGylation improve the efficacy of staphylokinase

**DOI:** 10.1186/s12934-017-0801-y

**Published:** 2017-11-14

**Authors:** Yanying Xu, Yueyuan Shi, Jianzhong Zhou, Wei Yang, Lei Bai, Shilei Wang, Xin Jin, Qiangsi Niu, Ailong Huang, Deqiang Wang

**Affiliations:** 1 Key Laboratory of Molecular Biology for Infectious Diseases (Ministry of Education), Institute for Viral Hepatitis, Department of Infectious Diseases, The Second Affiliated Hospital, Chongqing Medical University, YixueYuanlu-1, Chongqing, 400016 People’s Republic of China; 2 Collaborative Innovation Center for Diagnosis and Treatment of Infectious Diseases, Zhejiang University, Hangzhou, China; 3 Department of Cardiology, The First Affiliated Hospital of Chongqing Medical University, Chongqing, 400016 China; 4 Department of Laboratory Medicine, Chongqing Medical University, YixueYuanlu-1, Chongqing, 400016 People’s Republic of China; 5 Department of Laboratory Medicine, Hospital of Zhejiang, No. 12 Lingyin Road, Xihu District, Hangzhou, 310013 People’s Republic of China; 6 Department of Laboratory Medicine, The First Affiliated Hospital of Chongqing Medical University, Chongqing, 400016 China

**Keywords:** Staphylokinase, PEGylation, Immunogenicity

## Abstract

Staphylokinase (Sak) holds promise for use in thrombolytic therapy for acute myocardial infarction. However, its immunogenicity is a major disadvantage under clinical conditions. PEGylation has become a sophisticated method to decrease that immunogenicity. In this report, according predicted epitope from the active center, five residues, including Gly79, Leu82, Lys84, Ala97, and Arg104 have been mutant as cysteine for mono PEGylation, respectively. According to the relative immunogenicity of Sak or its PEGylation derivatives, the amount of specific anti-Sak IgG antibodies elicited by PEGylation proteins, including C79G, C82L, C84K, C97A, and C104R in BALB/c mice decreased by approximately 15–75% each. PEGylated Sak derivatives showed a decrease of up to 75% in the immune reactivity in PEG-Sak-C104R. Thrombelastography experiments showed that two PEG-conjugated derivatives, PEG-Sak-C97A (Ly30, 68.14 ± 2.51%) and PEG-Sak-C104R (Ly30, 66.49 ± 5.97%), the LY30 of PEG-Sak-C97A, and PEG-Sak-C104R produced values very similar to those of wild-type Sak. The fibrin plate assays showed the bioactivity of PEG-Sak-C104R to exhibit the most activity approximately as much as urokinase (diameter of halo pattern, 18.6 ± 1.06 mm) and tPA (diameter of halo pattern, 17.2 ± 0.49 mm). The Sak PEGylation derivative PEG-Sak-C104R was also selected for further in vivo activity experimentation. The thrombolytic ability of PEG-Sak-C104R is a little lower than wild-type Sak, whereas, this PEGylated protein retained high activity suitable for thrombolytic therapy. Collectively, with the in vivo and in vitro experiments, the present study suggests that site mutant PEGylation, PEG-Sak-C104R, is a suitable type of PEGylation for clinical applications. Further optimization would help maintain the bioactivity and decrease the immunogenicity of staphylokinase.

## Introduction

For patients with thrombotic diseases, thrombolytic therapy is the fastest and most economically viable means of treating blocked vessels in those with acute myocardial infarction [1]. Thrombus dissolution relies on activators of plasminogen reaching the surface of fibrin and surmounting the various inhibitors of clot lysis present in the plasma [2]. At present, there are three thrombolytic drugs, streptokinase, urokinase, and tissue plasminogen activator (t-PA), in clinical use [3, 4]. Streptokinase is less often used due to its adverse effect profile [5]. Urokinase, a serine protease composed of double chains joined by a disulfide bridge, has been the mainstay of clinical therapy for clearance of thrombi from arteriovenous grafts, but such agents have many clinical use restrictions, including major surgery, stroke, bleeding lesions, severe trauma and organ biopsies [6]. t-PA, a single-chain serine protease that binds to fibrin and then activates plasminogen to plasmin, is significantly more effective than urokinase with respect to restoring catheter patency, but the drug has a short half-life and is associated with risk of bleeding [7]. The cultivation of new thrombolytic agents with high effectiveness but low rates of side effects are urgently needed [1, 3, 4].

Staphylokinase (Sak), a 136-amino-acid profibrinolytic agent encoded by the Immune Evasion Cluster-positive prophages that are highly widespread in human *Staphylococcus aureus* (*S.* *aureus*) strains, has shown some promise for thrombolytic therapy of acute myocardial infarction [8, 9]. Additionally, staphylokinase is its capicity for neutralizing host antibacterial peptides whose binding may control its plasminogen activation properties [10]. Though it differs from endogenous activators like tPA and urokinase in its lack of proteolytic activity, Sak can induce plasminogen activation capability in human plasmin by establishing a 1:1 stoichiometric complex with its serine proteinase domain [11]. However, it has a major disadvantage: its immunogenicity [12, 13]. In this way, it is essential to reduce the immunogenicity to promote Sak for the effective clinical therapy.

One of the most biocompatible means of decreasing the immunogenicity of a protein is using hydrophilic PEG (polyethylene glycol) as macromolecular carrier [14]. PEGylation, covalent conjugation of PEG to proteins or peptides, is an established method of improving the therapeutic properties of protein drugs [15]. It can increase serum half-life and solubility and reduce immunogenicity, renal clearance, proteolytic sensitivity, and antigenicity, so providing a structural shield of protein surface from solvent environment [16, 17]. Current trends in protein drug PEGylation involve optimizing PEG-protein conjugates by site-directed monoPEGylation or designing new chemical linkages between PEG and the protein [18]. However, the effect of conjugation between PEG and protein on the PEGylated protein is still poorly understood.

So far several reports have focused on the Sak PEGylation, especially site-specific conjugation at N-terminus and C-terminus of Sak [19–22]. These Sak PEG derivatives with terminal modifications show 10–60% of the bioactivity of wild type Sak and decrease immunogenicity. In addition, four site monoPEGylation derivatives, including four lysines (Lys96, Lys102, Lys109, and Lys135) located in the core region also did not significantly reduce the immunogenicity [22]. There is currently an urgent need to investigate and develop new PEGylated schemes providing high bioactivity and low immunogenicity.

In the current study, we investigated the bioactivity and immunogenicity of predicted epitope PEGylated Sak in vivo and in vitro. Wild-type Sak does not contain any cysteine residues, suggesting that it provides a strategy to site PEGylation by mutation into cysteine. Consequently, according the predicted epitope from the active center, five residues, including Gly79, Leu82, Lys84, Ala97, and Arg104, have been mutated to cysteine for mono PEGylation, respectively. With the in vivo and in vitro experiments, our present study suggests that the PEGylation site mutant C104R may decrease the immunogenicity of Sak and maintain its thrombolysis activity.

## Materials and methods

### Animals

All rats and mice were nourished with sterile water and sterile standard pellet food ad libitum. The investigation was performed in accordance with the Guide for the Care and Use of Laboratory Animals published by the U.S. National Institutes of Health (NIH Publication No. 85–23, revised 1996). Animals were housed in groups of four to six under a 12-h light/dark cycle (lights on 6:00 a.m.), allowed food and water ad libitum, and acclimatized for 1 week. All animal work was carried out under appropriate licensing by the Committee on the Ethics in Animal Experimentation at Chongqing Medical University (Reference Number: 2016-0040).

### Construction of the wild-type and mutant Sak plasmids

To improve the efficiency of expression, the *Sak* (Accession Number WP_000920038) gene sequence was synthesized based on codon optimization. The optimized gene was subcloned into pET28a expression vector (Novagen, U.S.). According to active site (PDB code 2sak and 1bui) and epitope predication (ProPred-I, http://www.imtech.res.in/raghava/propred1/) [23, 24], five candidate PEGylation sites were selected and mutant to cysteine, including C79G, C82L, C84K, C97A, and C104R, respectively. The mutant Sak gene was amplified from the optimized sequence and verified by DNA sequencing. The resulting plasmid for the expression of wild type and mutant Sak in *E. coli* was named pET28a-Sak, pET28a-Sak-C79G, pET28a-Sak-C82L, pET28a-Sak-C84K, pET28a-Sak-C97A and pET28a-Sak-C104R, respectively.

### Expression and purification wild-type Sak and its site mutants

The recombinant expression plasmids were transformed into *E.* *coli* BL21 (DE3) cells for expression. Cultures of bacteria carrying the wild-type and mutant Sak plasmids were grown overnight in 20 ml LB medium by addition of 50 mg l^−1^ kanamycin. The bacteria were then used to inoculate 2 l medium and cultured at 310 K for about 3 h. The proteins were induced by adding 0.2 mM IPTG when the OD_600_ reached 0.4. Then the culture was allowed to grow at 293 K for a further 18–20 h before the cells were harvested by centrifugation. The cells from the 2 l culture were resuspended in lysis buffer (20 mM PBS pH 7.6, 300 mM NaCl) and lysed by sonication. The cell debris was removed by centrifugation at 15,000*g* for 30 min at 277 K. The recombinant protein in the supernatant was applied to a Ni^2+^–NTA affinity resin (Qiagen) column pre-equilibrated with lysis buffer. Nonspecifically bound proteins were washed from the column with washing buffer (20 mM PBS, 300 mM NaCl, 10 mM imidazole). Then the target proteins were eluted from the resin with 200 mM imidazole in PBS buffer. The eluted proteins were further purified by ion-exchange chromatography (DEAE, GE Healthcare) and separated using a linear gradient elution of NaCl (0–500 mM with PBS). Finally, the elution buffer containing wild-type or mutant Sak proteins were concentrated and buffer exchanged into the storage buffer (100 mM NaCl and 5 mM PBS, pH 7.6) by a 5 kDa cutoff Millipore Amicon concentrator (Millipore, Billerica, MA, U.S.) and stored at 193 K for subsequent studies.

### Preparation and purification of Sak PEGylated variants

Cysteine mutants of Sak were PEGylated using maleimide activated linear methoxy PEG of 10 KDa (Sigma) [25]. For the PEGylation reaction, the mutant proteins, including pET28a-Sak-C79G, pET28a-Sak-C82L, pET28a-Sak-C84K, pET28a-Sak-C97A and pET28a-Sak-C104R, were kept in 100 mM PBS buffer pH 7.6 with 100 mM NaCl, respectively. To this, about 5 molar excess of PEG reagent was added in reaction mixture which was allowed to gently stir at 277 K for 10 h, and the reaction was stopped by adding 1 mM of DTT.

PEGylated protein was separated from free PEG and the unreacted Sak mutants were separated by anion exchange chromatography on a DEAE Sepharose column (GE Healthcare) at 277 K. The reaction mixture was diluted 10–15 times with 25 mM PBS buffer pH 7.4 and loaded onto a column of DEAE Sepharose that had been pre-equilibrated with the same buffer. After washing with a 25 mM Sodium Phosphate pH 7.0 buffer, the bound protein was eluted using a linear salt gradient (0.1–0.3 M NaCl) in 25 mM PBS. Bi-PEGylated derivatives could not be cleanly separated from unreacted PEG by ion-exchange. For this reason, these samples were subjected to size exclusion chromatography on Superdex-75 (GE Healthcare) using 100 mM PBS buffer pH 7.6 containing NaCl (100 mM) for final purification.

### Immune-reactivity of Sak and its PEGylated variants

The relative immunogenicity of Sak or its cysteine mutants after PEGylation was examined using Sak anti-sera (polyclonal) raised in rabbits by an ELISA-based method [26]. A total number of 30 BALB/c mice (aged 8–10 weeks, weight 22–25 g) were averagely and randomly divided into six groups which were named as follows: wild type Sak, PEG-Sak-C79G, PEG-Sak-C82L, PEG-Sak-C84K, PEG-Sak-C97A, and PEG-Sak-C104R, respectively. Each mouse was injected with Sak or its PEGylated variants (300 μl) at protein concentration of 0.2 mg ml^−1^ in pre-cold buffer (100 mM PBS, pH 7.6) four times (0, 3, 10 and 17 days) by caudal vein. The mice bloods were collected on day 19 from the mice eye socket veins and centrifuged at 500 rpm for 15 min. The sera were collected and restored in − 80 °C for ELISA experiments.

The wild type Sak proteins (200 μl, 2 μg ml^−1^ in 10 mM PBS buffer, pH 7.6) were incubated in the 96-wells ELISA plates at 4 °C for 12 h. The plates were subsequently washed three times with 10 mM PBS buffer (pH 7.6). Then the serums, as prepared in the previous step, were diluted with 10 mM PBS buffer (pH 7.6) in four ratios, including 1:500, 1:1000, 1:2000, and 1:4000 respectively, and added in ELISA plates and incubated at 310 K for 2 h. The plates were emptied and washed in washing buffer (10 mM PBS and 0.1% Tween20, pH 7.6). The 100 μl horseradish peroxidase conjugated goat anti-mouse IgG (Sigma) was diluted in 10 mM PBS buffer (pH 7.6) with 1:5000 added in all wells of the plate and incubated at 310 K for 1 h. For the last step, the sample container was emptied and washed using washing buffer (10 mM PBS, 0.1% Tween 20, pH 7.6) five times, and then 100 μl of 3,3′,5,5′-tetramethylbenzidine (0.015%, w/v) (TMB, Sigma) was added to each well and incubated for 30 min at room temperature for coloration. To stop the coloration reaction, 25 μl 2 M H_2_SO_4_ was added to each well, and the color development was observed and recorded spectrometrically at 450 nm.

### Fibrin lysis of Sak and its PEGylated variants

To determine the fibrinolytic activity of Sak and its PEGylated variants, thrombolytic activity was assessed using fibrin plates as described previously with light modifications [27–30]. Briefly, the fibrin plates were produced by mixing 30 IU human fibrinogen (Sangon, China), 1 IU thrombin (Sangon, China) and 0.75 g plasminogen (Sangon, China), and then 15 ml of 1.0% agarose gel was added at ≈ 325 K. Subsequently, the fibrin plate was reinforced at 277 K for 1 h and equal diameter holes were punched in the plates. Then Sak and its PEGylated variants and control (5 k IU UK and 5 k IU tPA) were then loaded onto the plate, which was incubated at 310 K for 20 h. The activity of the unmodified Sak protein was then measured by the diameter of halo patterns.

### Thromboelastographic assays

Thromboelastography was performed by a TEG® 5000 Series Hemostasis Analyser System (Haemonetics, U.S.) to measure the viscoelastic properties of whole blood clots [31]. Then 500 ml of whole blood was pipetted into a customized vial containing wild type Sak and its PEGylated variants to a similar final concentration of 75 ng ml^−1^ Sak, and mixed by gentle inversion. The reaction mixture was then added to the TEG cup (Haemoscope Corporation) containing CaCl_2_ (20 μl, 200 mM); the samples (10 μl, 200 nM) in the TEG cup were processed using thromboelastogram software at 310 K for 1.5 h. The hemostatic parameters, such as split point (SP), reaction time (R-time), coagulation time (K-time), alpha angle (α), and maximum amplitude (MA), were recorded and analysed. SP reflects the initial fibrin formation, R is the initial clot formation time, both K time and α angle are direct indicators of thrombosis formation speed, and MA is indicative of the maximal strength of the clots. TEG experiments were repeated three times for each blood sample.

### In vivo thrombolysis activity

According to the results of immunogenicity and vitro experiments, a total of 30 SD rats (SD rats, weight 220–250 g) were randomly separated into two groups here called groups A and B, in which rats were injected with wild-type Sak and Sak PEG-conjugated derivative, respectively [32]. The thromboses in the carotid artery were processed by the galvanic stimulation instrument (XR-YLS-14B, Xinruan, China) under the following conditions: 2.0 mA, 1.25 mV, 10 min. The instrument could also provide an accurate monitoring of vascular blockage ratio. After the ratios were steadily maintained at 95–99% by galvanic stimulation, the rats were injected with drugs (wild type Sak and Sak PEG-conjugated derivative, respectively) at 2.0 mg kg^−1^ body weight. The exchanges of vascular blockage ratios were recorded and grouped into three grades, grade I (30%), II (30–70%), and III (> 70%).

### Statistical analysis

Results are here expressed as mean values ± SD. Statistical significance was tested using Student’s unpaired t test to compare two groups. The values of *P* < 0.05 and *P* < 0.01 were considered statistically significant and highly statistically significant, respectively.

## Results

### Construction of Sak mutants

**Fig. 1:**
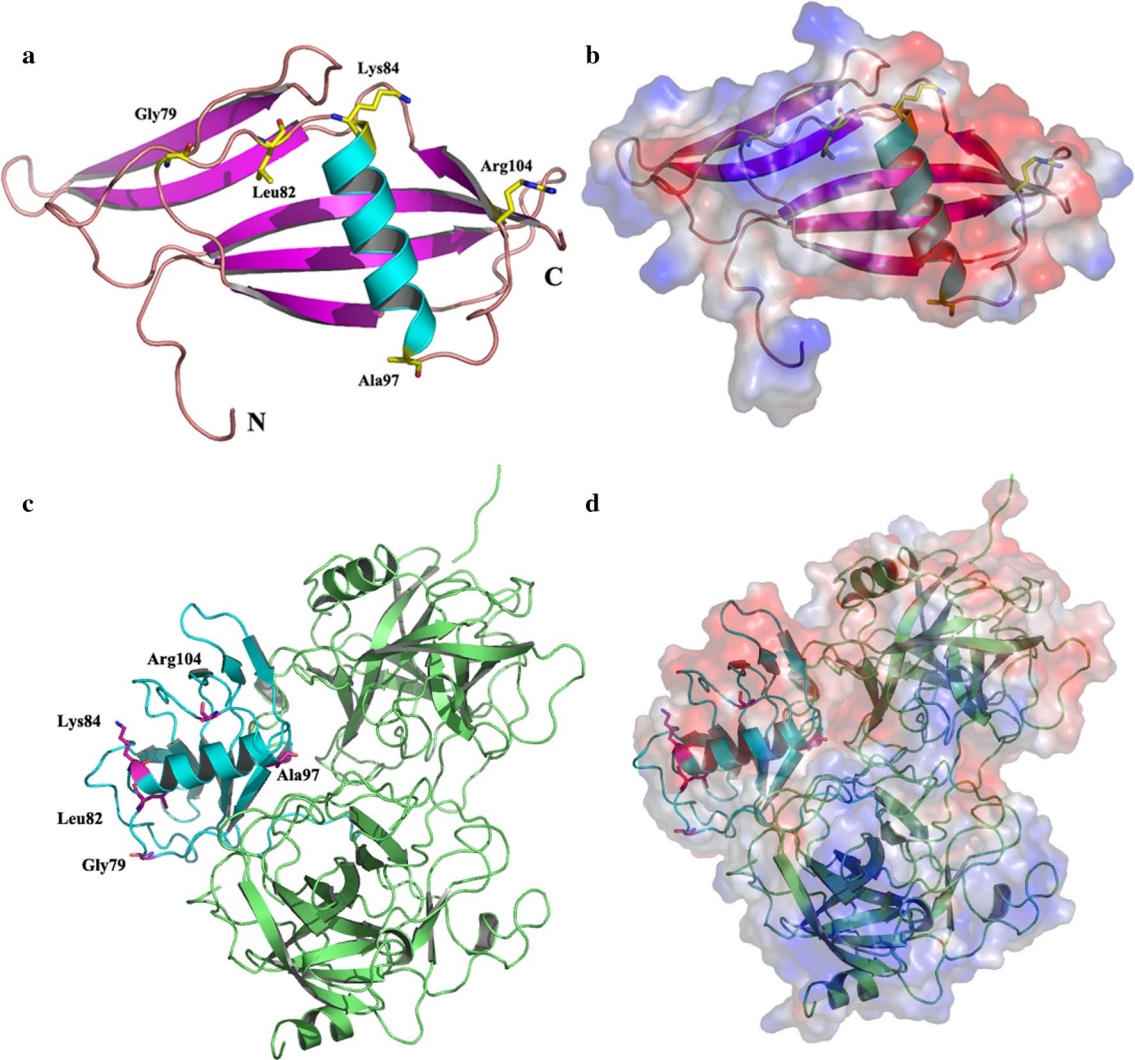
The site mutants selected for site PEGylation modified of Sak. **a** The five residues of Sak monomer selected for site PEGylation modified, Gly79, Leu82, Lys84, Ala97, and Arg104, were marked with sticks (PDB code 2sak). **b** Shown is the electrostatic potential surface of the monomer Sak (PDB code 2sak) in the same orientation as in **a**. **c** The five residues of Sak (Sak-μPl plasminogen complex) selected for site PEGylation modified, Gly79, Leu82, Lys84, Ala97, and Arg104, were marked with sticks (PDB code 1bui). **d** Shown is the electrostatic potential surface of Sak-μPl plasminogen complex (PDB code 1bui) in the same orientation as in **c**. Saturating Red indicates A < − 10 kiloteslase/e, and saturating blue indicates A > 10 kiloteslase/e, T = 293K

The wild-type Sak sequence from *S. aureus* is completely devoid of cysteine, which provides an opportunity for site-specific PEGylation after introducing cysteines at sites based on epitope, activity and other structural considerations deduced from its three-dimensional structure. According to epitope prediction and three-dimensional structure analysis, five residues, including Gly79, Leu82, Lys84, Ala97, and Arg104, were selected. These are found principally on exposed parts of the surface of the protein and it has been shown that mutations in this region do not significantly impede biological activity of Sak (Fig. 1) [23, 24]. Once the active mono-PEG substituted derivatives were identified, double-(bi) and triple-(tri) PEG substituted Sak derivatives were constructed and studied for bioactivity and immunogenic characteristics.

### Preparation of Sak and its PEGylation mutants

**Fig. 2:**
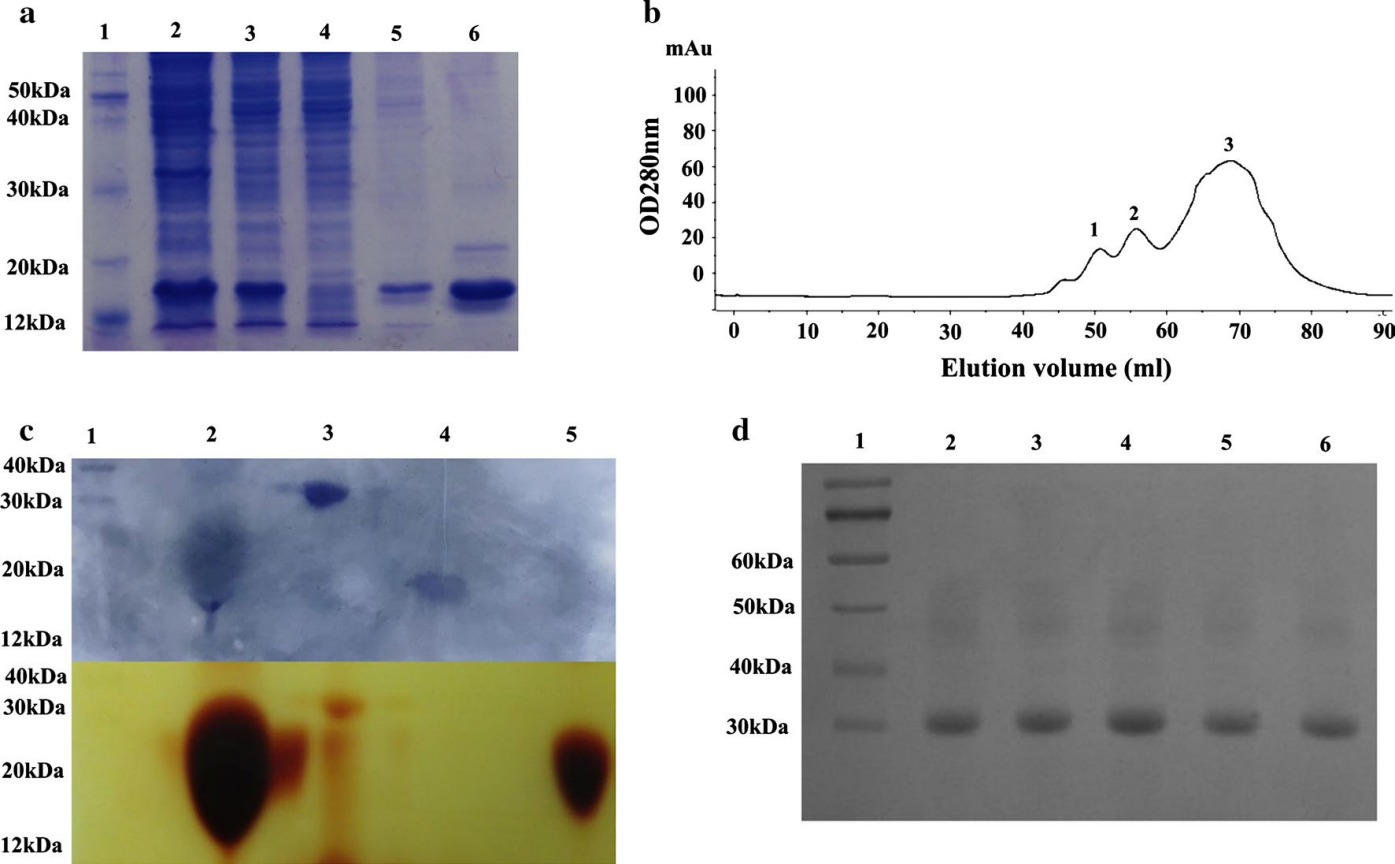
Preparation of Sak and its PEGylation mutants. **a** The Sak-cys protein (Sak-C79G) was purified by Ni^2+^-NTA affinity chromatography. Lane 1: protein marker; lane 2: pellet; lane 3: supernatant; lane 4: flow through; lane 5: elution with washing buffer; lane 6: elution with elution buffer. **b** Size exclusion gel filtration chromatography of Sak using the Superdex 200 column. **c** Figures with Coomassie blue staining (upper part) and identification of iodine staining (lower part) are combined together. Lane 1: protein marker; lane 2: reaction mixtures; lane 3: elution of peak 1; lane4: elution of peak 2; lane 5: elution of peak 3. **d** SDS-PAGE analysis of the PEG-Sak proteins. Lane 1: protein marker; lane 2: PEG-Sak-C79G; lane 3: PEG-Sak-C82L; lane 4: PEG-Sak-C84K; lane 5: PEG-Sak-C97A; lane 6: PEG-Sak-C104R

All of the cysteine-substituted mutants of the *Sak* gene with codon optimization were cloned in pET-28a vector and expressed in *E.* *coli (*BL21-DE3) cells for soluble expression. The optimized *Sak* gene exhibited efficient soluble expression with over 30–50 mg l^−1^ LB medium. As shown in Fig. 2a, both mutants were purified in a two-step process involving Ni^2+^-NTA affinity and anion-exchange chromatography. The purification yields of the mutants, including Sak-C79G, Sak-C82L, Sak-C84K, Sak-C97A, and Sak-C104R and wild type Sak were prepared to a purity of about 90%.

In order to minor minimize conformational changes and active influence, 10 kDa PEG-maleimide (PEG-Mal) was selected as a PEGylation reagent [25]. In all cases, the predominant reaction product was the expected mono-PEGylated protein. The PEGylated-Sak conjugates could be separated from un-reacted Sak and from free PEG by size-exclusion chromatography (Superdex 200) and were recovered as a distinctly sharp peak in the chromatogram (Fig. 2b, c). Iodine staining and western blotting confirmed that this band (≈ 20 kDa) was detected by an anti-HisTag monoclonal antibody, suggesting that the purified band represents Sak PEG-conjugated mutants (unpublished data). As shown in Fig. 2d, all of the PEG-conjugated derivatives, including PEG-Sak-C79G, PEG-Sak-C82L, PEG-Sak-C84K, PEG-Sak-C97A, and PEG-Sak-C104R, exhibited similar migration corresponding to the expected position exhibited by a standard with a MW of 20 KDa position during SDS-PAGE, with about 85% purity, respectively.

### Immune reactivity of PEGylation derivatives

**Fig. 3:**
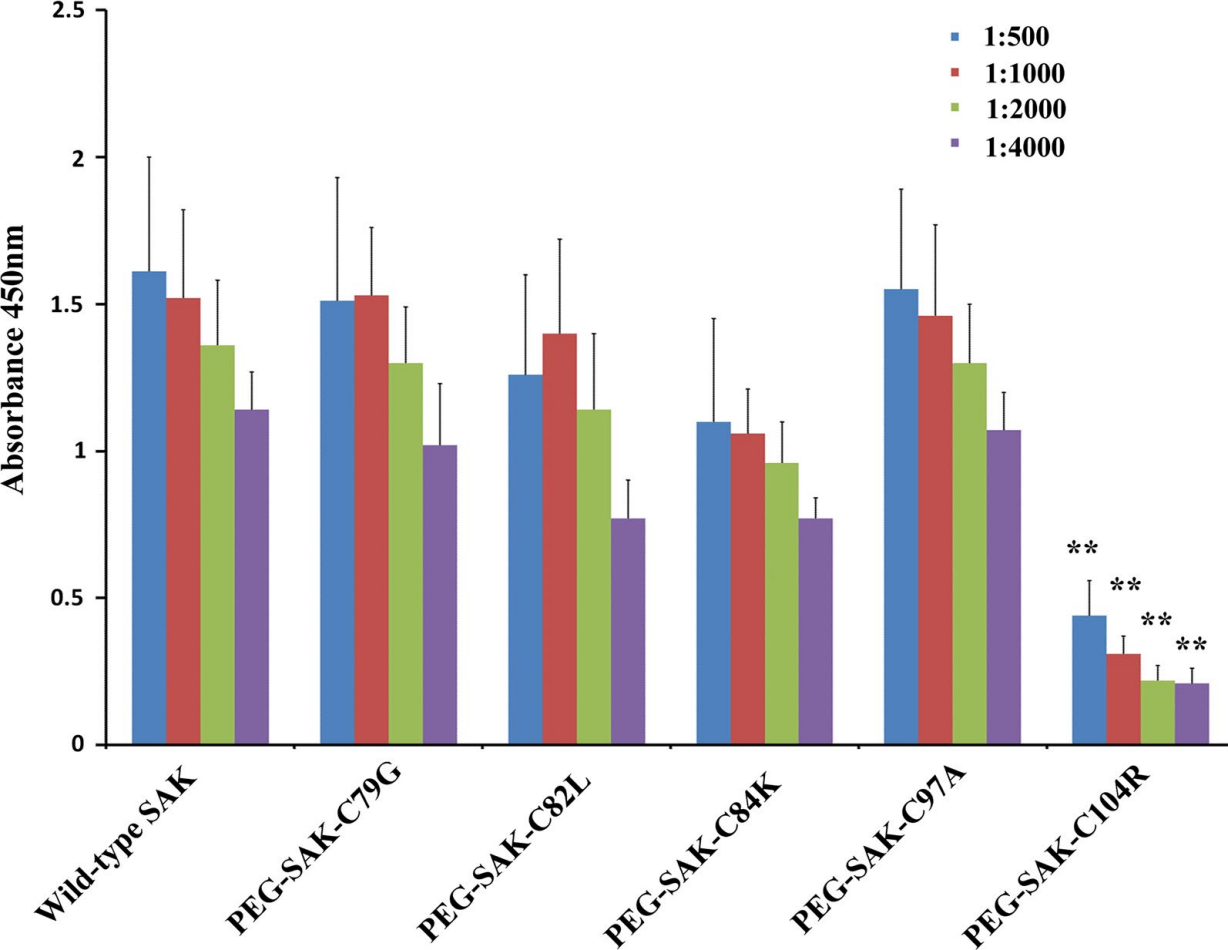
Anti-Sak IgG levels of the unmodified Sak and PEG-Sak-Cys. The anti-Sak igG immunoresponse to the unmodified Sak and peg-Sak-cys in serum dilution of 1:500, 1:1000, 1:2000 and 1:4000, respectively. (**P < 0.01 compared with unmodified Sak)

Sak, which shows potential for the treatment of thrombolysis, has a major disadvantage in its immunogenicity. This can have some dramatic effects on treatment and lead to adverse side reactions, such as thrombocytopenia and aplastic anemia anaphylactic responses. Nevertheless, PEGylation can improve protein efficacy by reducing proteolytic sensitivity and immunogenicity. The immunogenicity of the PEGylated SAKs was determined in this way. As shown in Fig. 3, investigation of the immunogenicity of Sak PEGylation derivatives showed that the amount of specific anti-Sak IgG antibodies elicited by PEGylation proteins, including PEG-Sak-C79G, PEG-Sak-C82L, PEG-Sak-C84K, PEG-Sak-C97A, and PEG-Sak-C104R, in BALB/c mice was approximately 15–75% lower than in wild-type Sak. Among these PEGylated Sak derivatives, PEG-Sak-C104R was found to decrease immune reactivity by as much as 75%, suggesting that residue 104 of Sak might the most advantageous site for PEGylation.

### In vitro thrombolytic activity

**Fig. 4:**
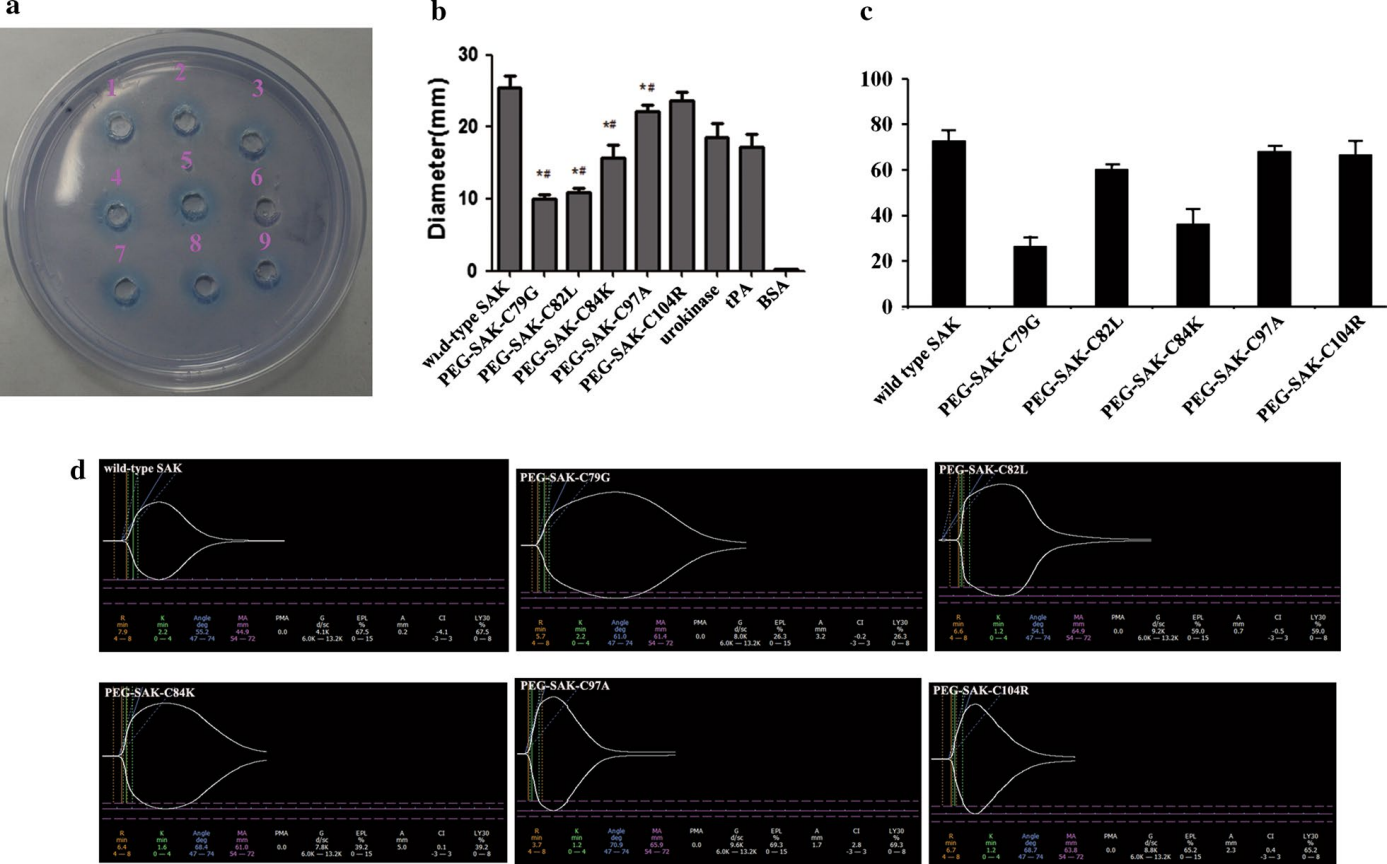
The thrombolysis activity had measured with the fibrin plate and TEG analyzer. **a** Thrombolytic activity of Sak determined by fibrin plate. Stoma 1: 0.1 mg PEG-Sak-C79G; Stoma 2: 0.1 mg PEG-Sak-C82L; Stoma 3: 0.1 mg PEG-Sak-C84K; Stoma 4: 0.1 mg PEG-Sak-C97A; Stoma 5: 0.1 mg PEG-Sak-C104R; Stoma 6: 0.1 mg BSA; Stoma 7: 5.0 × 10^3^ IU unmodified Sak; Stoma 8: 5.0 × 10^3^ IU urokinase; Stoma 9: 5.0 × 10^3^ IU tPA. **b** Diameters of the lysis dots on the fibrin plate from at four independent evaluations. (Values are the mean ± SD, n = 3; *P < 0.05 compared with wild-type Sak. #*P* < 0.05 compared with urokinase and tPA). **c** Thrombolysis activity of Sak determined by TEG analyzer. Ly30, the percent lysis 30 min after maximum amplitude. (Values are the mean ± SD, n = 10; *P < 0.05 compared with wild-type Sak). **d** Test results of the wild-type and five PEGylated SAKs by TEG analyzer

The plasminogen activator protein Sak achieves its function primarily by forming a plasminogen active complex with plasmin to prevent biofilm formation. Consequently, fibrin plate assays were used to assess whether the Sak and its PEGylated variants were actively folded. Sak PEGylated variant proteins exhibit different levels of thrombolytic activity. As shown in Fig. 4a, b, the mono PEGylated variants, including PEG-Sak-C79G, PEG-Sak-C82L, PEG-Sak-C84K, PEG-Sak-C97A and PEG-Sak-C104R, exhibited thrombolytic activity slightly lower than that of wild-type Sak (diameter of halo pattern, 25.4 ± 1.21 mm), suggesting this mutant and PEGylation interfered a little with the activity of the plasminogen activator protein. Among these five PEG-conjugated derivatives, the activity of PEG-Sak-C104R exhibited the most activity, much more than that of urokinase (diameter of halo pattern, 18.6 ± 1.06 mm) and tPA (diameter of halo pattern, 17.2 ± 0.49 mm), indicating that this derivatives has the potential for clinical application.

The fibrinolytic activity of Sak and its PEG-conjugated derivatives were further assessed using a TEG analyzer. LY30 (percent lysis 30 min after maximum strength of clot) functions as one of the main hemostatic parameters of fibrinolysis system activity. As shown in Fig. 4c, d, two PEG-conjugated derivatives, PEG-Sak-C82L (Ly30, 60.3 ± 2.25%), PEG-Sak-C97A (Ly30, 68.14 ± 2.51%), and PEG-Sak-C104R (Ly30, 66.49 ± 2.51%), exhibited levels of activity similar to that of wild type Sak (72.8 ± 4.66%). The activity of PEG-Sak-C79G (Ly30, 26.48 ± 4.66%) and PEG-Sak-C84K (Ly30, 36.29 ± 6.52%) were much lower than those of the other three derivatives.

### In vivo thrombolytic activity

**Fig. 5:**
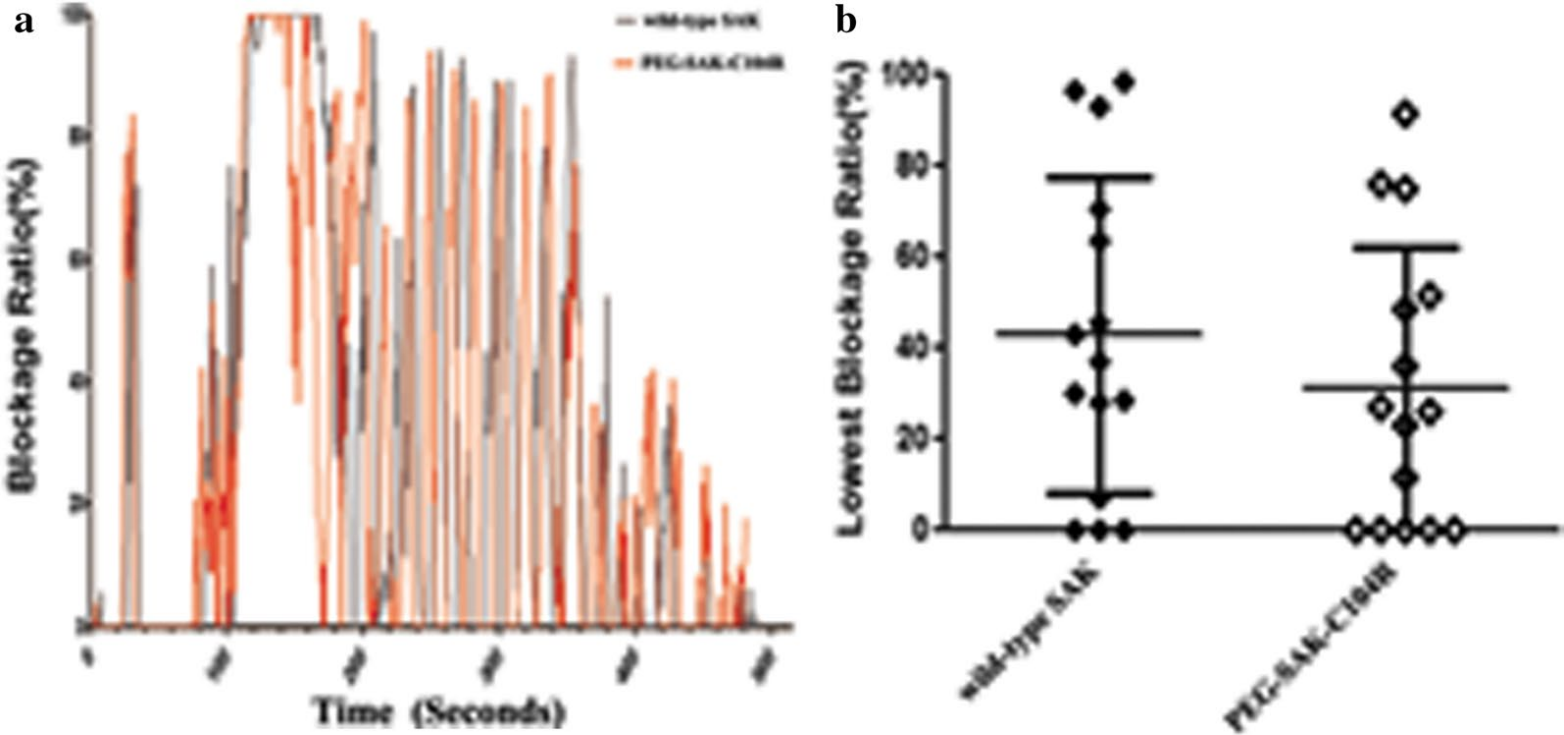
In vivo thrombolytic activity by SD rats. The SD rats that reached the lowest blockage ratios after injection of the samples. **a** Test results of four cases (wild-type Sak, No. 4, 7 and 12 of PEG-Sak-C104R) by carotid artery blood flow with high blockage ratios (> 70%). **b**, **c** Embolism unblocking in SD rats treated with the wild-type Sak and PEG-Sak-C104R

On account of the results of previous in vitro activity experiments, among the five PEGylation derivatives, PEG-Sak-C97A and PEG-Sak-C104R exhibited the most thrombolysis activity. In addition, PEG-Sak-C104R induced the least immunogenicity in mice of any anti-Sak. For this reason PEG-Sak-C104R was selected as the most potential Sak PEGylation derivatives for further in vivo activity experiments. According to a relative dose response test, the study had chosen 2 mg kg^−1^ body weight as the experimental dose (data not shown). As shown in Fig. 5a, b, the PEG-Sak-C104R showed an activity level approximately equal to that of the wild-type Sak protein. In control group, the thrombus dissolved 5–8 min after injection of wild-type Sak. Among these mice, nearly 45% showed reductions in thrombus size of 70, and 40% of the mice showed a 30–70% blockage ratio. Only about 13.4% did not experience thrombolysis or any other effect. Besides, the PEG-Sak-C104R groups showed effectiveness levels similar to the fore groups, and the rate of low (< 30%), middle (30–70%), and high blockage ratios (> 70%) were 26.7, 53.3, and 20%, respectively. In general, the thrombolytic ability of PEG-Sak-C104R proteins was slightly lower than in wild-type Sak, whereas, this PEGylated protein still showed pronounced activity suitable for thrombolytic therapy.

## Discussion

In this report, according to the predicted epitope and crystal structure of Sak, five specific sites of PEGylation were here prepared. This led to variant thrombolytic activity and immunogenicity. Relative to other wild type and PEGylation Sak proteins, PEG-Sak-C104R showed the most pronounced ability to retain the thrombolytic activity and decrease the immunogenicity of the original.

Sak, a candidate drug for thrombolytic therapy of acute myocardial infarction, has relatively high immunogenicity and short plasma half-life [12, 13]. It should therefore be administered by continuous infusion. PEGylation, an effective method of improving the pharmacological profiles of Sak, acts by decreasing immunogenicity [24, 25]. Recently, PEGylation has been performed with conjugation at amino, thiol, hydroxyl, and amide groups, and there are more options available for the PEGylation of proteins [33]. Specifically it works by conjugating amino acid residues, including arginine, histidine, glutamine or, cysteine. The wild type Sak from *S.* *aureus* is completely devoid of cysteine, which provides a particularly effective method of site-specific PEGylation of Sak by linking “maleylation” to cysteine residues based on predicted epitope and bioactivity [25]. Epitopes of antigen could be conveniently identified by the receptors on the surface of lymphocytes to activate the lymphocytes that cause the immune response. The epitope of Sak might be a suitable candidate site for PEGylation to decrease immunogenicity [25]. Because the PEG polymer is directly conjugated with Sak by covalent bonds, the location for PEGylation is immensely important because these derivatives might markedly influence the bioactivity of the modified molecule [33]. By combining the epitope predicted by ProPred-I and activity location deduced by crystal structure (PDB code 2sak and 1bui), five candidate residues, including Gly79, Leu82, Lys84, Ala97, and Arg104, were selected (Fig. 1). These five residues located at two loop regions connecting by one α-helix, which were far from the substrate (μPl plasminogen) binding site, indicating that PEGylation at these sites could maintain thrombolysis activity and decrease the immunogenicity [23, 24].

PEGylation of therapeutic proteins often gives rise to substantial reductions in bioactivity, presumably due to the dimensional shielding effect of PEG molecule, which prevents the protein from coming into contact with the substrate [14, 33]. According to evaluate the bioactivity of these five PEGylation derivatives, including PEG-Sak-C79G, PEG-Sak-C82L, PEG-Sak-C84K, PEG-Sak-C97A, and PEG-Sak-C104R, two in vitro bioactive inspections, fibrin lysis, and thromboelastography active assay, were used here. As shown in Fig. 4, the specific activity of two mono-PEG derivatives, PEG-Sak-C97A and PEG-Sak-C104R derivatives PEGylated at the epitope II, was more than 90% that of native Sak. However, under similar reaction conditions, three derivatives, PEG-Sak-C79G, PEG-Sak-C82L, and PEG-Sak-C84K, demonstrated activity markedly more sluggish than that of wild-type Sak. According to previous research, covalent attachment of PEG molecule on Sak at residue Asp109 did not affect its thrombolytic properties, which is consistent with our experiments, suggesting that the polymer molecule PEG at residues Ala97 and Lys104, similar to the residue Asp109, does not interfere with substrate interaction with Sak at its bioactive site [22]. Instead, results showed that three derivatives, PEG-Sak-C79G, PEG-Sak-C82L, and PEG-Sak-C84K, exhibited much less activity than wild-type Sak, suggesting that the large PEG groups introduced at epitope I (residues 79–84) synergistically interfered with the interaction between Sak and plasminogen [24]. However, the steric shielding effect of PEG is not adequately understood, and further experimentation is required to indicate the steric shielding effect of PEG on the therapeutic protein through experimental and molecular simulation analyses.

The immune-reactivity of the PEGylated-Sak derivatives and wild-type Sak was ascertained by competitive ELISA using polyclonal anti-sera against Sak generated in mice. Irrespective of site of conjugation of PEG group, three of the mono-PEGylations of Sak, including PEG-Sak-C82L, PEG-Sak-C84K, and PEG-Sak-C104R, led to significant overall reduction in the immune reactivity (Fig. 3). PEG-Sak-C104R demonstrated more than 80% decrease in the immune reactivity from wild type, but PEG-Sak-C82L and PEG-Sak-C84K exhibited only about 30% decrease in immunogenicity, whereas, PEG-Sak-C79G and PEG-Sak-C97A showed immune reactivity similar to that of the wild-type Sak. In the past, the principal antigenic epitopes in Sak assessed using a phage-displayed library of Sak variants was selected for mutants that escape binding to an affinity matrix derived from patient-specific polyclonal anti-Sak Abs [34]. According to the phage-displayed library, the region consisted by residues 102–104 may be a relevant epitope. This is very consistent with our results of immunogenicity experiments. Additionally, our fibrin lysis and Thrombelastography active assay have confirmed that PEG-Sak-C104R maintained the similar thrombolytic activity to that of the native Sak (Fig. 4). Similarly, we conducted thrombolytic experiments in rats to further verify the thrombolysis of PEG-Sak-C104R (Fig. 5), which maintained efficient thrombolysis to these electrode thrombosis models in rats. Collectively, the PEG-Sak-C104R owned the great potentiality qualified the clinical thrombolysis therapy.

In conclusion, according the predicted epitope from the active site, five residues, including Gly79, Leu82, Lys84, Ala97, and Arg104, have been mutant as cysteine for mono PEGylation. According to the immune reactivity, PEG-Sak-C104R is associated with significantly less immunogenicity than wild-type Sak and other epitope PEGylated-Sak derivatives, including PEG-Sak-C79G, PEG-Sak-C82L, PEG-Sak-C84K, and PEG-Sak-C97A. Additionally, thrombolytic activity (in vivo and in vitro), PEG-Sak-C104R showed much more bioactivity than other PEGylated derivatives. Collectively, our reports provide a solid data that PEG-Sak-C104R is potential candidate for further optimization to retain bioactivity and decrease immunogenicity for clinical application.
